# Understandings, attitudes, practices and responses to GHB overdose among GHB consumers

**DOI:** 10.1186/s12954-023-00857-z

**Published:** 2023-09-02

**Authors:** Jack Freestone, Nadine Ezard, Adam Bourne, Jonathan Brett, Darren M. Roberts, Mohamed Hammoud, Anthony Nedanoski, Garrett Prestage, Krista J. Siefried

**Affiliations:** 1 The Kirby Institute, University of New South Wales Sydney, Wallace Wurth Building, High St, Kensington, NSW 2052 Australia; 2 ACON, 414 Elizabeth Street, Surry Hills, NSW 2010 Australia; 3 National Centre for Clinical Research on Emerging Drugs, c/o the University of New South Wales, Sydney, 2052 Australia; 4 Alcohol and Drug Service, St Vincent’s Hospital Sydney, Darlinghurst, 2010 Australia; 5 The National Drug and Alcohol Research Centre (NDARC), The University of New South Wales, Sydney, 2052 Australia; 6 Australian Research Centre for Sex Health and Society, La Trobe University, Building NR6., Bundoora, VIC 3086 Australia; 7 Edith Collins Centre, Drug Health Services, Royal Prince Alfred Hospital, Sydney, NSW 2050 Australia

**Keywords:** Gamma hydroxybutyrate, Overdose, Pre-hospital, Emergency medical services, Harm reduction, LGBTQ +, Sexualized drug use

## Abstract

**Background:**

Gamma-hydroxybutyrate (GHB) is used at disproportionately high rates within sexuality and gender diverse communities and carries a high risk of overdose. GHB overdose can result in death. Internationally, recent increases in GHB overdoses have been observed. Coronial reviews of GHB-related death highlight the pivotal roles that bystanders to GHB overdose play in preventing fatality. No research has examined, in detail, how bystanders respond to GHB overdose. This qualitative study was conducted among people who use GHB and explored how they responded upon witnessing a GHB overdose experienced by someone else.

**Methods:**

Interviews were conducted with 31 sexuality and gender diverse Australian residents reporting three or more occasions of GHB use in the previous 12 months. Participants were asked questions about witnessed GHB overdose, their actions and decision-making processes throughout overdose. Data were analysed thematically.

**Results:**

Participants described witnessing GHB overdose, commonly in private settings involving sexualized GHB use. Variable definitions of GHB overdose were reported, ranging from GHB-induced symptoms of distress to comatose intoxication. Drastic actions to keep someone alert and responsive post-GHB ingestion were reported; these included the administration of stimulant substances and citrus. Decisions to call or not call for emergency medical services (EMS) were influenced by many circumstantial variables. In most instances, an EMS call was resisted and response practices deviated from established first aid protocols.

**Conclusions:**

GHB overdose prevention and response training programs targeting people who use GHB are urgently required. These education interventions ought to address inaccuracies that inform street remedies for GHB overdose, teach people how to safely check breathing and response, promote basic first aid principles and address barriers to contacting EMS.

## Background

Gamma-hydroxybutyrate (GHB) and its precursors gamma butyrolactone (GBL) and 1,4-butanediol (1,4BD) are potent central nervous system depressants when consumed at higher dosages, which carry a high risk for overdose and severe toxicity [1]. GHB overdose is common among people who use GHB [2] and may result in hospitalisation [3] or death [4, 5]. To our knowledge, no research has been published that examines, in detail, how people who use GHB respond upon witnessing a GHB overdose in others. People who use GHB, who also observe GHB overdose in others, can intervene to save lives [5], and efforts to educate GHB consumers in overdose response must be informed by studies that explore common GHB overdose response practices.

Studies from Australia, the United Kingdom, the USA and the Netherlands have found a low population level prevalence of current GHB use, ranging from 0.1 to 1.3% [2]. However, a higher prevalence of GHB use is recorded among specific subpopulations. These populations include people attending nightclubs [6, 7], gay and bisexual men (GBM) engaged in sexualised drug use, also known as chemsex or party-and-play [8, 9], and one review notes the popular use of GHB in sexual settings across LGBTQ + communities [10].

The term ‘GHB overdose’ is broad and the literature indicates that it is variably defined and understood by researchers [11] and consumers alike [12, 13]. Commonly differentiated are the experiences of falling asleep after using GHB, in contrast to GHB-induced coma [11]. The term GHB overdose may also describe GHB-induced cold sweats, seizures, vomiting, involuntary muscle spasms (dystonia or myoclonus) or other distressing symptoms, with the terminology of overdose used as a descriptor regardless of whether these symptoms are followed by loss of consciousness [8, 14, 15].

The experience of either personal or witnessed GHB overdose is common among GHB consumers [12]. A systematic review of GHB using populations observed a lifetime incidence of GHB-induced coma ranging between 25 and 69% [2]. GHB overdose is so common because of its steep dose response curve, variations in the strength and purity of GHB, intraindividual variations in tolerance and contraindications with other central nervous system depressants such as alcohol [1, 2]. Witnessed GHB overdose is rarely assessed in quantitative studies; however, participants in qualitative studies among people who use GHB often report witnessing GHB overdose [16–18]. These studies do not focus on actions taken following a witnessed GHB overdose.

There is evidence that GHB overdoses may be increasing internationally and in Australia. Analysis of European emergency department monitoring data observed that GHB was the fourth most common drug involved in drug related emergency department presentations between 2014 and 2017 [19]. A coronial review from London noted an increase in GHB-related overdose deaths between 2011 and 2015 and attributed this increase to the increased use of GHB in the context of sex [20, 21]. A study from the Australian state of Victoria documented a 147% increase in GHB-related ambulance attendances between 2012 and 2018 [22]. In New South Wales, a total of 1298 GHB-related emergency department presentations were recorded between July 2019 and June 2020, representing a sevenfold increase since 2011 [23]. However, GHB-related hospitalisation is likely to be underestimated. This is because GHB is not routinely screened for in toxicological analysis [24] and is rapidly excreted [25].

However, evidence demonstrates that most GHB overdoses are not attended by emergency medical services (EMS) [17, 26]. In a qualitative study conducted among 45 GHB consumers recruited via outreach at community venues and snowball sampling, 142 separate occasions of GHB overdose were recorded and only three of these occasions were attended by medical professionals [17].

While first aid principles universally promote calling for EMS to assist when people are found to be non-responsive [27], people who use drugs experience several barriers to calling EMS, these barriers include a fear of legal consequences and cost [28]. Much research addressing consumers’ attitudes and actions regarding EMS attendance has focussed on people who use opiates [28, 29] but to date, there has not been a focus on GHB. Several studies report that GHB consumers may feel that GHB overdose does not pose a serious risk, which may, in turn, reduce their likelihood of calling EMS [3, 7, 30–32]. However, a variety of other considerations may affect how people who use GHB make decisions regarding EMS attendance.

Although there is no antidote to reverse the cardiac or respiratory depression associated with GHB overdose [33], bystanders to GHB overdose may mitigate fatality risks by keeping a person’s airways clear and promptly calling EMS [1, 5]. GHB has been characterised as a club drug [34, 35]; however, GHB is also commonly used with other people [17] in private and domestic settings [16]. Private settings may provide suitable conditions for applying harm reduction measures to prevent GHB overdose (such as timing or measuring GHB doses) [12]. However, domestic settings do not afford people the support of trained venue staff or onsite medical teams [30]. As has been observed in coronial reviews of GHB-related mortality, the use of GHB in private settings accentuates the important role that bystanders play in responding to a witnessed GHB overdose [4, 5, 20, 26].

In the opiate overdose literature, studies outlining street remedies for overdose have informed lifesaving opiate overdose response interventions [36, 37]. Street remedies for GHB overdose have only been briefly reported in academic literature [18, 30] and data to inform essential GHB overdose response education initiatives is lacking.

This paper explores common understandings, attitudes, practices and responses to GHB overdose among GHB consumers. Our data address voluntary GHB consumption and do not reflect experiences of GHB facilitated sexual assault [38]. Our intention is to examine how GHB overdose is understood by people who use GHB, illuminate helpful and safe overdose response practices alongside unhelpful, misguided and potentially harmful practices. We explore how consumers understand risks associated with GHB in accordance with their perceptions around overdose severity and we characterise how these perceptions impact their response practices. This paper draws on data collected from interviews among people who use GHB from lesbian, gay, bisexual, transgender and queer (LGBTQ +) communities. We focus on LGBTQ + populations as studies have reported increased GHB use and GHB-related overdose deaths among LGBTQ + communities [4, 8, 20].

## Methods

### Participant eligibility and recruitment

We interviewed 31 participants, to be eligible, participants needed to currently live in Australia, identify as LGBTQ +, report GHB consumption on three or more occasions in the previous 12 months, understand and communicate in English, consent to have their interview audio recorded and to be transcribed. Participants were recruited via advertising on community organisations’ social media. Snowball recruitment methods were also used.

### Interview process

Interviews were conducted between March and October 2021. On average, interviews lasted approximately 55 min. Interviews were conducted by the first author, a researcher with extensive experience within the drug and alcohol services sector and expertise in GHB-related harm reduction education. Some interviews were conducted in person at the offices of a community organisation; however, the majority were conducted via video teleconferencing facilities.

Interviews were semistructured and conducted with the assistance of an interview guide. Our interview guide was developed by the entire authorship team alongside staff from partner community organisations and people with lived and living experience of GHB use. The interview guide was piloted on an initial three participants and then collaboratively iterated by authors JF, KJS, AN, GP, AB and NE.

Interviews commenced with the collection of demographic and behavioral characteristics. All participants were then asked questions about the contexts of their GHB use, safety and well-being during use, experiences of GHB associated pleasures, harms, sexual practices, overdose experiences and overdose response. Participants were asked questions about whether they had witnessed GHB overdose, and those who reported that they had, were then asked to describe the overdose event in detail. Where participants reported witnessing multiple GHB overdoses, participants were asked to describe one overdose event in detail. Participants were asked how they responded when witnessing the GHB overdose. Probing and follow-up questions pertaining to the setting of overdose, actions taken to ensure wellbeing and decisions to call or not call for EMS.

Participants were reimbursed with a $50 grocery voucher for their time.

### Data analysis

Interviews were transcribed verbatim. NVivo® (QSR International) was used to facilitate data analysis. All data were anonymised at analysis and participant names are reported as pseudonyms. Data were analysed using a thematic framework approach [39].

Full transcripts were reviewed by the first author JF. Participants’ accounts, experiences, and perceptions were coded through an unrestricted and inductive process. Codes were then collaboratively arranged into emerging themes by JF and KJS. Full transcripts were reviewed and discussed with KJS and in collaboration with co-authors KJS, AB and GP. Throughout discussions within the authorship team, the coding framework was iteratively revised. Data relating to experiences and perceptions of GHB overdose were descriptively analysed, and results were reviewed by all authors.

## Results

31 participants were interviewed as part of this study (Table 1).

**Table 1:** Number of participants by sexual orientation, gender identity, location, age and frequency of GHB use

*Sexual orientation*	
Gay	10
Queer	10
Bi +	7
Lesbian	2
Heterosexual	2
*Gender identity*	
Cisgender man	14
Nonbinary	8
Cisgender woman	6
Transgender woman	2
Transgender man	1
*Location*	
Sydney	12
Melbourne	12
Perth	4
Regional	2
Brisbane	1
*Age*	
18–24	6
25–34	12
35–44	9
45–54	2
55–64	2
*GHB use frequency in previous 12 months*	
Three or four times a week	3
Weekly	1
Fortnightly	5
Monthly	8
Every two months	3
Every three months	4
Sporadically > 3 times a year	6

Ten participants reported no personal experience of overdose, three reported experiencing symptoms of distress after using GHB without then experiencing sleep or loss of consciousness, and 18 reported either involuntarily falling asleep after using GHB or losing consciousness after using GHB. Five participants reported a personal experience of GHB-related hospitalization, and 17 reported responding to someone else who had either fallen asleep or lost consciousness after using GHB.

Three key theme areas emerged from the analysis of our data that related to the experiences and perceptions of GHB overdose. These were [1] understandings of GHB overdose, [2] reported responses to GHB overdose and [3] considerations around calling EMS.

Most participants in our study reported personally experiencing or witnessing a GHB overdose. Overdoses were described and understood as being variably severe, ranging from inducing distressing symptoms such as vomiting, to comatose intoxication. Overdoses were most often narrated as occurring in private and sexualised settings where multiple other drugs were also consumed. Sometimes participant’s responses to a witnessed GHB overdose appeared to align with established first aid protocols [40], other times, participants deviated from standard first aid protocols and exercised judgements informed by prior lived experience, peer attitudes, beliefs and group norms or other circumstantial variables that may have precluded a more standard response, for example, calling EMS.

### Understandings of GHB overdose

Participants used the terminology of ‘blow out’ or ‘GHB drop’ to connote the range of experiences or symptoms that they associated with GHB overdose, an experience that was characterised in accordance with either symptoms of distress, GHB-induced sleep with responsiveness, or GHB-induced sleep unresponsive to pain or sound stimuli.

Symptoms of GHB-related distress were in some instances described as preceding a potential loss of consciousness. Symptoms described as egregious, unpleasant and alarming were said to include vomiting, sweating, convulsions, energised agitation or aggression, panic, incessant talking and facial contortions.

“I’ve seen it when I’ve been out, and it’s generally been like they’ve been convulsing, or they’ve been frothing from the mouth a bit”

Jose, cisgender man, gay, 30s.

The early signs and symptoms of GHB overdose appeared to be easily recognisable to participants and were said to include incessant animated talking, facial contortions, muscle spasms, cold sweats, vomiting, sleepiness, and displays of manic aggression.

“Everything just animated and urgent. But it could be trying to claw their way up the stairs and get onto the street. Like they can feel something’s gonna happen to them and they’ve clicked on that it’s got to do with the G”

Sabine, non-binary, queer, 50s.

Sabine speaks of the heightened activity that may be associated with GHB overdose. GHB is a drug with stimulant, disinhibitory and depressant effects [41] and the unpredictable nature of GHB overdose was narrated in contrast to the overdose experience of other central nervous system depressants such as opiates.

The experience of GHB-induced sleep was often described as overdose but also narrated as harmless and transient, requiring a friend or bystander to monitor without further intervention, as is reflected by Jim:

“I have been in a situation where people have passed out… One was a guy who was just a hook-up… I stayed there for an hour or two, and he was just fast asleep. So, I left a note and left … ‘Cause he hadn’t passed out or anything; he had just fallen asleep”

Jim, cisgender man, gay, 50s.

Participants appeared most concerned about GHB overdose in which friends, acquaintances or sexual partners were said to have experienced an unrousable sleep after using GHB. GHB overdose resulting in comatose intoxication was articulated by some (not all) as the threshold at which to call EMS. This is noted by Emily:

“If someone is responsive, maybe they’re not talking but they will open their eyes if someone says their name…That for me is like, “Okay, this person’s had too much but it’s not at a level yet where I think they’re in danger of dying,” as opposed to if someone is passed out, completely unresponsive. You know, there’s shallow breathing, that kind of stuff. That for me is like… “There’s nothing we can do here except call an ambulance.”

Emily, trans woman, queer, 20s.

### Reported responses to GHB Overdose

Nearly all accounts of witnessed GHB overdose were reported in circumstances where participants responding were also using GHB or other drugs such as crystal methamphetamine, cocaine or ketamine. Self-intoxication while responding to overdose was broadly perceived as not impeding an appropriate response.

“I can have a pharmacy in my system but, as soon as like something bad is happening, my adrenaline or whatever levels just go… That’s to one side: this needs to happen.”

Jermaine, cisgender man, pansexual, 30s.

GHB overdose was narrated as occurring over several stages; response actions associated with each of these stages are outlined in Table 2.

**Table 2:** Participants’ reported responses to managing diverse symptoms associated with stages of GHB overdose, and first aid recommended actions

Participants report			First aid recommended actions
Stage	Response	Actions taken by bystanders	
Initial symptoms of distress	Preparing for safety	Checking in, e.g., asking if ok. Providing water to sip. Moving to a quiet environment with less stimulus	Assess for dangers, remove hazards and mitigate risks associated with falling Check on other medical conditions Ask what substances have been taken and when Ask for emergency contact information if a deterioration occurs and hospital attendance is required
Urge to sleep or near loss of lucidity	Dosing stimulants	Dosing stimulants such as cocaine or crystal methamphetamine (perceived to help retain alertness responsiveness)	Increases intoxication, may cause stimulant toxicity, stimulants not an antidote to GHB overdose.*
	Providing citrus	Providing someone with a drink of orange juice Putting a lemon or lime wedge in mouth	No evidence
	Keeping someone alert	Standing under a shower Biting nipple Rubbing sternum Shouting Shaking, slapping & hitting Splashing Punching throat	First aid principles promote checking responsiveness by squeezing shoulder or speaking loudly.** Call emergency medical services if someone is not responsive.** Biting, shaking, slapping, hitting, standing someone upright, forcing someone into a shower or punching may risk accident and injury
GHB-induced sleep (responsive and non-responsive)	Checking for responsiveness	Waking someone with pain or sound stimulus Asking someone to say their name Asking someone to squeeze hand	First aid principles promote checking responsiveness by squeezing shoulder or speaking loudly.**
	Observing	Sitting with someone for a duration of hours Placing someone in the next room and intermittently monitoring	Call emergency medical services if someone is not responsive and do not leave someone who is non-responsive unmonitored.**
	Assessing breathing	Monitoring for unusual breathing sounds, patterns, or faint breathing	Call emergency medical services if someone is not responsive, look listen and feel for normal breathing.** If non-responsive and not normal breathing, then first aid recommendations are to call EMS and start CPR**
	Managing airways	Putting into the recovery position	Call emergency medical services if someone is not responsive, assess breathing and place in the recovery position.** To manage airway open mouth and check for foreign material. If foreign material is present roll the patient onto their side and clear the airway. If there is no foreign material, leave the patient in the position found, and open the airway by tilting the head back with a chin lift
	Ensuring comfort	Putting someone in a spare bedroom or safer location Ensuring body has not contorted into a potentially injurious position	Call emergency medical services if someone is not responsive, assess breathing and place in the recovery position. Remove environmental hazards and do not leave the person unmonitored**
	Collaboration	Lifting the deadweight of a body to a separate location, Discussing decision to call or not call for ambulance	Call emergency medical services if someone is not responsive. Only move non-responsive person if they are in immediate danger Assess breathing and place in the recovery position.**
	Getting help	Driving someone to hospital Calling an ambulance / emergency medical services Getting a ride-share to hospital	Call emergency medical services if someone is not responsive.**

*Centre for Disease Control. Polysubstance Use Facts. from http://www.cdc.gov/stopoverdose/polysubstance-use/index.html

**St John Ambulance Australia. (2017). *Emergency First Aid* (Fourth ed.) from: http://www.stjohnvic.com.au/media/1932/pfa1d.pdf

***St John Ambulance Australia. (2022) First Aid Fact Sheet DRABBCD Action Plan from stjohn.org.au/assets/uploads/fact%20sheets/english/Fact%20sheets_DRSABCD.pdf

Narrations of GHB overdose response were often characterised by an anxious urgency to keep someone awake and responsive. Actions taken to keep someone responsive are reflected in the *“Urge to sleep or near loss of lucidity”* section of Table 2. In most circumstances, a combination of these actions was applied.

“I’ve always said to people, “Make sure that person stays awake. Stop, don’t let them fall asleep because you don’t know how asleep they’re falling,” ‘cause it could be just, you know, having a little nap or it could be passing out, stop breathing, whatever, you know.”

Ivan, cisgender male, bisexual, 40s.

Like Ivan, most participants engaged in efforts to keep someone awake after using GHB. However, some appeared comfortable with letting people “sleep it off”. Some regarded GHB sleep as a normal and transient occurrence, requiring monitoring of breathing and airway management but no further intervention. Participant’s willingness to let someone “sleep it off” appeared to be influenced by factors relating to polydrug use (whether the person experiencing overdose had consumed alcohol or any other depressants alongside GHB), familiarity with the person, and the participant’s prior personal experiences of GHB-induced sleep.

“Like it depends on who it is that is taking it and if you’ve, you know what they’re like when they’ve had G. And you go, “Okay, that’s alright. He’s just sleeping it off.”

Craig, cisgender man, gay, 50s.

“So there’s looking after them in that way where I’m not necessarily worried about any contraindications with any other alcohol or drugs they’ve had, that might lead to any overt situation or respiratory shutdown… because I know what they’ve had and I know who they are… I can generally say I’ve put them to bed pretty safely. Obviously in that situation I’ll keep an eye on them.”

Michelle, transwoman, heterosexual, 30s.

Michelle highlights her practice of keeping an eye on someone who is asleep after using GHB. Central to many accounts of monitoring throughout GHB sleep was the management of airways and use of the recovery position. Assessments of breathing were reported to involve looking and listening for breath to assess whether or not breathing remained “normal”. The word normal was predominantly defined in relation to the absence of abnormalities in breathing patterns, overt sounds of snoring or breathing that was determined to be faint, this is reflected in Emily’s quote above.

Above, Michelle and Craig described circumstances in which they felt that occasional monitoring and allowing someone to sleep after GHB use was appropriate. Below, Michelle speaks of contrasting circumstances of GHB overdose that required more active management.

“There have been people who’ve had accidentally had massive doses who I’ve had to literally, you know, spend the next eight hours pulling their tongue out of their throat so they don’t choke on it, constantly rolling them back into recovery position and force-feeding them orange juice, and trying to keep ambulance officers on speed dial without actually calling them because the places we were and the things we were doing were potentially not the ideal place to have paramedics through or places where other people wouldn’t allow us to have paramedics there. But regardless this is a thing that I’ve never actually trained for apart from lived experience.”

Michelle, transwoman, heterosexual, 30s.

Michelle references the influence of place on the decision to call EMS. Upon probing, Michelle did not wish to speak more about how place-based considerations influence EMS calls, however, some other participants reflected hesitations to calling EMS to other people’s residences and in settings where other drugs were present or where people were often having sex.

Above, Michelle acknowledges that she has never received formal overdose response training and this sentiment was echoed by other participants. One participant referred to receipt of first aid training, and three spoke about receipt of drug education, harm reduction and overdose response training via community organisations. Otherwise, practices around overdose response appeared to be informed by lived experience and in some circumstances online resources or network education among fellow GHB consumers.

### When to seek help? Consideration for calling EMS

Calling EMS was narrated by participants as a serious decision associated with negative consequences such as cost and potentially distressing hospitalisation. Table 3 outlines the considerations that discouraged or motivated decisions to call emergency medical services.

**Table 3:** Reported considerations that discouraged or motivated decisions to call emergency medical services in response to GHB overdose

	Factor	Considerations
Discouraging factors	Cost	Making someone else liable to pay an ambulance fee, disputes arising related to ambulance attendance fees
	Hospitalisation distress	A concern for experiences of stigma against people who use drugs in hospital settings, distress and confusion associated with waking up in hospital
	Legal ramifications	Belief that police will co-attend GHB overdose, fear of onwards legal ramifications
	Overdose environment	Not feeling empowered to call an ambulance to another person’s residence, locations where other drugs present, sex occurring
	Prior experience of GHB overdose	Prior experience of non-fatal GHB overdose; sense that overdose is transient and not harmful
Dually discouraging and motivating factors	Confidence to manage GHB overdose	Knowledge and prior experience of managing GHB overdose impacted anxiety around overdose response and willingness to call for an ambulance, those less experienced with GHB overdose were more willing to call EMS, by contrast to those with more experience of GHB overdose
	Other respondents	Peer pressure not to call an ambulance or group collaboration and support for EMS call
	Breathing assessment	Breathing deemed ‘normal’ prevented ambulance call, by contrast faint, shallow or abnormal breathing increased likelihood of EMS call
	Responsive assessment	Ability to gain a response via pain or sound stimuli prevented ambulance call, no response increased likelihood of EMS call
Motivators	Accidental GHB ingestion	Rapid loss of responsiveness after accidental ingestion of a large dose of GHB motivated decision to call EMS on all reported occasions

Several participants reported that the fear of ‘getting into trouble’ was a barrier to calling an ambulance, believing that police always attend when paramedics are called to assist with overdose. Similar barriers have been observed in the broader drug overdose response literature [42–44].

When deciding whether to call EMS, participants weighed the potential negative consequences of EMS attendance against the risk of someone sustaining serious injury or dying. The act of not calling EMS was sometimes described as an act of care motivated by a desire to protect the financial, emotional and social well-being of those experiencing overdose.

“I would expect it if I was in that position and I needed an ambulance, that someone would call one... But I would expect, if I was in a position where I just had like a ml too much and I was like, kind of like just falling asleep, that I didn’t wake up in hospital because… you know, when you start mixing drug world with real world, real world looks at you and goes, “You idiot. You loser. You this. You that.”

Benjamin, cisgender man, bisexual, 30s.

Benjamin’s concerns about experiencing stigma associated with drug use were echoed by other participants.

Benjamin also draws a distinction between an overdose that does not require EMS, using the words “a ml too much” and “just falling asleep” and a more serious overdose that would require an ambulance. Categorization of overdose as either harmless and transient by comparison to serious and requiring EMS appeared to hinge on the assessment of someone’s breathing and ability to respond to pain or sound stimuli as is demonstrated by both Mark and Allan.

“He just fell down. He was snoring. He was sweating, sweating profusely but it was like a cold sweat. It was just, yeah, you couldn’t, you couldn’t wake him up and an ambulance had to be called.”

Mark, cisgender man, gay, 30s.

By comparison, Allan resisted calling EMS in a situation where someone he was caring for started to demonstrate an ability to respond to pain and sound stimuli.

“It was getting to the stage where we thought, “Another three minutes and we will call an ambulance.” And just right then it started to really become a lot more focused. I mean he was dropping in and out. It wasn’t like he was totally unconscious.”

Allan, cisgender man, gay, 60s.

Participants near-unanimously stated that if they observed someone experiencing faint or shallow breathing, or a complete inability to respond, EMS would be called. Yet in some accounts, even when participants had concerns about someone’s ability to respond they did not call EMS. Below, Martin describes why he did not call EMS throughout a GHB overdose when an acquaintance was not responsive.

“You said you don’t know why you didn’t call an ambulance. Do you have any theories about why?”

“Group think? Based on what I knew, those people had more experience in dealing with GHB than me. You know, I’m newer to it, if you like. They were kind of taking charge as to what to do. Like they were like, “No, he needs to sit down.” “No, he needs to stand up.” “No, if we put cold water on him, he’ll be fine. If I just massage his chest, he’ll kind of like come …” I’m thinking, “Okay, they’ve clearly been through this before and know the things to do, to fix it.”

Martin, cisgender man, gay, 30s.

Martin reflects on different levels of expertise and confidence in managing an overdose which in turn influenced their willingness to call EMS. It appeared that participants who had frequently witnessed GHB overdose were more confident in managing GHB overdose without calling for EMS, as demonstrated by Alina.

“I haven’t called an ambulance ‘cause I can do a lot of the tests myself. I’m pretty good at testing if they’re rousable… So, I feel I take on a bit more risk in that regard. So, I guess what I would treat as an overdose in terms of like practically as an overdose where I would like to call an ambulance or freak out is if it was, it would be someone that I couldn’t rouse”

Alina, genderqueer, queer, 20s.

In three circumstances participants witnessed someone accidentally ingesting a large dose of GHB after mistaking GHB for another drinking liquid. In each of these circumstances participants observed a rapid onset of GHB overdose and promptly called EMS**.**

## Discussion

To our knowledge, no studies have specifically addressed bystander response to GHB overdose, despite a prevalent experience of GHB overdose among those who consume GHB [2, 11]. Our data outline a range of factors that influence overdose response and highlight many possible opportunities for education.

Some reported responses to GHB overdose were tailored to overdose symptoms and appeared to be safe. These included the removal of stimulus and hazards, use of the recovery position to manage airways, and assessment of breathing. However, several responses warrant further attention in education initiatives.

Remedies believed to help with GHB overdose included the administration of citrus or other stimulant drugs, drastic attempts to retain responsiveness such as punching, biting, slapping and the use of cold showers. The drastic nature of actions highlights potential risks for aspiration, injury or (in instances where stimulants were dosed) additional intoxication [40, 45].

Most participants reported that they learned how to respond to a GHB overdose via lived experience and peer education rather than formal training. Several overdose response education and training programs have been delivered for people who use opiates [46]. Opiate overdose prevention programs often focus on the administration of naloxone, CPR and promote calling EMS [47]. Evaluations of these training programs have demonstrated their acceptability and feasibility [48], and it has been reported that people who use opiates are motivated to protect their peers and intervene to prevent fatality upon witnessing overdose [49]. While there is no antidote to reverse the effects of GHB overdose [33], like opiate overdose, GHB overdose can be fatal [26] and the immediate response of those who witness GHB overdose may prevent death. Our findings demonstrate that people who use GHB are willing and eager to intervene to protect the well-being of their peers upon witnessing overdose. However, our findings and the findings of coronial reviews of GHB-related mortality [5, 20, 24, 26] indicate a need to test the acceptability and feasibility of delivering overdose response training programs to people who use GHB. The objective of this training program would be to dismantle unhelpful or unsafe street remedies for GHB overdose while teaching people how to manage airways, monitor breathing and call for EMS. The delivery of such a training program online may enable broad geographic reach, anonymous and cost effective attendance.

In our study, GHB overdose was most commonly narrated as occurring in private settings, within closed social and sexual networks of fellow GHB consumers. For this reason, we believe that GHB overdose response training programs should ideally target people who use GHB rather than targeting, for example, young people who attend nightclubs or sexuality and gender diverse communities more broadly.

The belief that GHB-induced sleep is often transient and harmless has been extensively documented [26, 30, 31]. This belief was reflected in our findings; our participants widely reported their opinion that it was reasonable to check for responsiveness, monitor breathing, and then let someone sleep after using GHB. This act requires attention in harm reduction education and in GHB overdose response training programs for people who use GHB because GHB-induced sleep presents a fatality risk [26].

Participants in our study reported several practical and circumstantial barriers to calling EMS; where practical, these barriers ought to be addressed. Barriers led some participants to narrate the act of calling an ambulance in response to GHB overdose, interpreted to be a ‘GHB sleep’ as not just unnecessary but also potentially harmful. Participants were often willing to spend several hours actively monitoring and supporting someone experiencing GHB overdose to ensure their safety and avoid calling an ambulance. In some circumstances the act of not calling an ambulance was regarded as an act of care. Cultures of care have long been documented within networks of sexuality and gender diverse people who use drugs [50]. While it is encouraging to observe the ethos of these cultures enacted by our participants, work to reduce the perceptibly negative consequences of EMS attendance such as legal consequences, cost and the experience of stigma and discrimination within hospital settings may shift attitudes toward calling EMS and increase the safety of people who use GHB.

## Conclusions

Our findings support the imperative of delivering overdose response education programs targeting people who use GHB. We postulate that opiate overdose response training programs may present a useful framework for similar GHB overdose response training programs. Education programs must highlight the dangers associated with GHB-induced sleep and support people who use GHB to learn how to safely and accurately check for responsiveness and assess airway and breathing. Education programs must promote calling EMS and support GHB consumers to make informed decisions about when to call EMS. Where possible, barriers to calling EMS should be addressed. Strategies to mitigate barriers to calling EMS may address ambulance costs, policies around police co-attendance, and promote inclusive care for people who use drugs in hospital settings.

### Limitations

Our findings need to be interpreted with regard to limitations. Our data were collected as part of a broad study about contexts, cultures and practices of GHB use, as such experiences of overdose, overdose response and hospitalization were not a singular focus. Further research specifically focussed on GHB is required to explore strategies used to assess consumer approaches to checking for responsiveness, managing airways and assessing breathing and vitals in more detail. No one in our sample reported current daily GHB use, as such our data do not reflect the experiences of those who may be dependent on GHB. Our data do not address experiences associated with GHB facilitated sexual assault and no one in our sample reported an experience of GHB-related fatality.

## Data Availability

The datasets used and/or analyzed during the current study are available from the corresponding author on reasonable request.

## Abbreviations

CPR: Cardiopulmonary Resuscitation
EMS: Emergency medical services
GHB: Gamma-hydroxybutyrate
LGBTQ +: Lesbian, gay, bisexual, transgender and queer
